# Predictive value of peripheral blood inflammatory markers for epilepsy occurrence in traumatic brain injury patients

**DOI:** 10.1186/s42494-025-00226-2

**Published:** 2025-06-26

**Authors:** Sheng-xue Wang, Qiang Zi, Yu-xuan Li, Wang Guo, Yu-hao Chu, Xue-ping Yang, Yun Li

**Affiliations:** 1 Clinical Medical School, Dali University, Dali, 671000 China; 2 Department of Neurology, The First Affiliated Hospital of Dali University, Dali, 671000 China

**Keywords:** Epilepsy, Traumatic brain injury, Neutrophil-to-lymphocyte ratio, Peripheral blood inflammatory markers

## Abstract

**Background:**

Post-traumatic epilepsy (PTE) is characterised by recurrent epileptic seizures following traumatic brain injury (TBI). PTE has a high incidence and leads to significant disability rates, posing a substantial socioeconomic burden. This study aimed to evaluate the predictive value of peripheral blood inflammatory markers—including neutrophils, lymphocytes, platelets, the neutrophil-to-lymphocyte ratio (NLR), and the platelet-to-lymphocyte ratio (PLR)—for seizure risk in patients with TBI.

**Methods:**

This investigation involved the enrollment of patients admitted to the Department of Neurology/Surgery at the First Affiliated Hospital of Dali University in Yunnan Province, China, spanning the period from January 2020 to May 2023. Our cohort comprised 138 individuals with PTE, 150 with TBI, 142 with epilepsy of unknown origin, and 130 healthy controls (HC). We retrospectively analysed demographic characteristics and peripheral blood cell inflammation markers for all participants.

**Results:**

1. In the PTE group, both neutrophil count and NLR exhibited higher levels compared to the TBI group, the epilepsy of unknown origin group, and the HC group. Conversely, the lymphocyte count, platelet count, and PLR in the group were lower in contrast to the TBI group, the epilepsy of unknown origin group, and the HC group. 2. Neutrophil count, lymphocyte count, platelet count, NLR, and PLR were significantly different between the PTE and TBI, PTE and HC groups (*P *< 0.05). Marked distinctions were detected in neutrophil count, platelet count, NLR, and PLR between the PTE group and the epilepsy of unknown origin group (*P *< 0.05). 3. Furthermore, our multivariate linear regression analysis unveiled that the TBI site (temporal lobe) (*P *< 0.05), the severity of TBI (mild, moderate, severe) (*P *< 0.05), and surgical intervention (*P *< 0.05) are the risk factors affecting the peripheral blood inflammation indicators. 4. Finally, the ROC analysis produced an AUC of 0.908 for neutrophil levels (cut-off: 4.05, sensitivity: 0.783, specificity: 0.992) and an AUC of 0.960 for NLR (cut-off: 2.945, sensitivity: 0.797, specificity: 0.992).

**Conclusions:**

Both neutrophil count and the NLR were significantly increased in the PTE group, suggesting that these parameters may serve as predictors of epilepsy development in TBI patients

**Supplementary Information:**

The online version contains supplementary material available at 10.1186/s42494-025-00226-2.

## Background

Traumatic brain injury (TBI) ensues from a substantial external force impacting the head, resulting in cerebral damage [1]. The clinical severity of TBI is categorised based on the Glasgow Coma Score (GCS) as mild (GCS 14–15), moderate (9–13), or severe (3–8) [2]. Epilepsy, a chronic neurological disorder, afflicts over 70 million individuals globally, imposing a considerable burden on both society and individuals [3]. TBI constitutes a significant risk factor for epilepsy development [4]. It is estimated that around 5.48 million people suffer from a severe TBI each year (73 cases per 100,000) [5]. TBI stands as the primary cause of symptomatic seizures in adults [6]. Post-traumatic epilepsy (PTE) constitutes 10–20% of symptomatic epilepsy cases [7]. Subsequent to a traumatic brain injury, seizures are classified as immediate (< 24 h), early (1–7 days), or late (> 1 week) events [8]. Seizures not only contribute to heightened morbidity and mortality shortly after TBI but also emerge as a substantial cause of death in the years following the injury [9]. Prior studies have suggested that PTE is linked to an increased risk of mortality [10]and a diminished functional prognosis [11].

Presently, no specific therapeutic interventions or preventive strategies exist for PTE [12, 13]. Despite prophylactic treatment, 4–53% of TBI patients still have chronic seizures [14]. The underlying mechanism of PTE remains incompletely elucidated [15]. Contemporary research suggests that neuroinflammation contributes to the development of PTE, while oxidative stress and mitochondrial dysfunction may further exacerbate its initiation and progression [16]. Pertinent investigations have suggested that individuals with TBI with brain contusions and associated inflammation have an elevated risk of epilepsy development compared to those without inflammation [17].

The neutrophil-to-lymphocyte ratio (NLR) and platelet-to-lymphocyte ratio (PLR) have emerged as novel inflammatory markers linked to the progression and prognosis of diverse neurological disorders, encompassing myasthenia gravis, Guillain–Barre syndrome, glioma, Alzheimer's disease, and acute coronary syndrome [18–22]. Chen et al. unveiled that NLR independently prognosticates the severity of TBI, with elevated NLR values correlating with diminished GCS scores [23]. Similarly, Li et al. identified PLR levels as an autonomous risk determinant for short-term mortality in individuals with moderate to severe TBI, highlighting substantial diagnostic potential [24]. Aslan and Cevik determined that each incremental unit in NLR elevates the likelihood of seizures by a factor of 1.239 [25]. Güneş et al. established an association between seizures and PLR, indicating that 1-unit increase in PLR escalates the risk of seizures by 1.95 times [26].

We built on the above studies to explore the correlation with PTE by evaluating the levels of neutrophils, lymphocytes, platelets, NLR and PLR in the serum of patients, providing important insights for the early identification and treatment of PTE.

## Methods

### Study design

Using a case–control design, this study is a single-centre retrospective study including patients admitted to the Neurology/surgical department of the First Affiliated Hospital of Dali University, Yunnan Province, China, from January 2020 to May 2023.

### Participant

The study encompassed 138 participants diagnosed with PTE, 150 with TBI, and 142 with epilepsy of unknown origin. Additionally, 130 healthy controls (HC) individuals constituted the control group. For the diagnosis of PTE, a minimum of two seizures subsequent to TBI was mandatory [27].

The inclusion criteria for PTE patients comprised: (1) Explicit evidence of traumatic brain lesion or a verified history of TBI through imaging like head CT or MRI. (2) No occurrence of seizures before the brain trauma. (3) Seizures arising following a TBI. (4) Diagnosis aligning with the 2014 International League against Epilepsy (ILAE) criteria. (5) Prophylactic use of patients preventing antiseizure medications (ASMs).

PTE patient exclusion criteria comprised: (1) Family history of epilepsy. (2) All epilepsy except for the PTE. (3) Presence of concurrent infectious, tumour-related, autoimmune, or other diseases.

TBI is characterised as damage to the brain resulting from impact, blow, or head vibration, or a penetrating head injury causing a disturbance in normal brain function. TBI is categorised into closed (non-penetrating) or open (penetrating) based on the injury's nature [1].

In this study, the inclusion criteria for TBI patients comprised: (1) Evident traumatic brain lesion or a history of TBI confirmed through imaging, like head CT or MRI. (2) No occurrence of seizures before and after the brain trauma. (3) Patients with TBI underwent routine blood tests within 24 h after admission.

Exclusion criteria for TBI patients involved: (1) Patients with brain trauma combined with epilepsy or epileptic seizures. (2) Presence of concurrent infectious, tumour-related, autoimmune, or other diseases.

Participants with epilepsy of unknown origin in this study fulfilled the diagnostic criteria established by the International League against Epilepsy (ILAE) in 2014. Patients with epilepsy had routine blood tests within 24 h of admission. The HC cohort comprised individuals without any medical conditions, selected from the physical examination centre.

### Procedure

In this investigation, we compiled demographic and clinical data for each participant, encompassing information such as gender, age, smoking, and alcohol consumption history. Among patients with TBI, we compiled data on the affected brain regions, including the frontal, temporal, parietal, and occipital lobes. We also documented the causes of TBI, such as traffic accidents, falls, or head strikes. Furthermore, we acquired information from head CT scans and GCS scores. Hematological data, comprising monocyte, lymphocyte, and platelet counts, were obtained for every participant. The NLR and PLR were computed using these hematological data. For TBI patients experiencing a second seizure, routine blood tests (including NLR and PLR) have been completed within 24 h. If epilepsy manifested after TBI, the individual was categorised into the PTE group. Those without epilepsy development were assigned to the TBI group. The identical data acquisition process was implemented for individuals in the control group.

### Statistical analysis

In conducting data analysis, SPSS version 25.0 software was employed. Qualitative data were succinctly represented as frequency and percentage, while quantitative data were articulated as mean ± standard deviations. For evaluating disparities between groups, chi-square tests were utilised for qualitative variables, and independent Kruskal–Wallis tests were deployed for quantitative variables. Kruskal–Wallis tests were also employed for pairwise comparisons among multiple groups. To scrutinize pertinent influencing factors, multivariate linear regression was implemented. Additionally, ROC curve analysis was executed to forecast the incidence. Statistical significance was established at a *P*-value less than 0.05 (*P* < 0.05).

## Results

### Characteristics of participants

Overall, 560 eligible patients were included in the present study. Among them, the mean age in the PTE group was 44.67 ± 15.64 years, 44.88 ± 17.18 years in the TBI group, 41.44 ± 17.64 years in the epilepsy of unknown origin, and 44.05 ± 17.28 years in the HC group. A total of 307 (54.82%) patients were male and 253 (45.18%) were female; 241 (43.04%) had a smoking history, and 319 (56.96%) had no smoking history. Concerning alcohol, 174 (31.07%) had a history of drinking, and 386 (68.93%) had no history of drinking.

Substantial distinctions emerged in smoking (*P* = 0.008) across the four groups. Concerning the site of brain trauma, notable variances surfaced in the frontal lobe (*P* = 0.032) and temporal lobe (*P* = 0.001). Furthermore, there were notable dissimilarities in the severity of TBI, with mild (*P* = 0.037), moderate (*P* = 0.043), and severe (*P* = 0.001) TBI cases exhibiting disparities among the groups. The prevalence of surgical operation also manifested significant differences among the groups (*P* = 0.001). Age (*P* = 0.314), sex (*P* = 0.860), alcohol use (*P* = 0.176), site of brain trauma (parietal lobe [*P* = 0.705] and occipital lobe [*P* = 0.428]), and cause of brain trauma (traffic accidents [*P* = 0.118], falls [*P* = 0.503], head strike [*P* = 0.265]) showed no significant differences (*P* > 0.05). The characteristics of the study population are delineated in Table 1.

**Table 1:** Characteristics of the study population

Characteristic	PTE (*n* = 138)	TBI (*n* = 150)	Epilepsy of unknown origin (*n* = 142)	Control (*n* = 130)	*P*
Age (year), mean ± SD	44.67 ± 15.64	44.88 ± 17.18	41.44 ± 17.64	44.05 ± 17.28	0.314^*^
Sex, *n* (%)					0.860^**^
Male	76 (55.1%)	78 (52.0%)	79 (55.6%)	74 (56.9%)	
Female	62 (44.9%)	72 (48.0%)	63 (44.4%)	56 (43.1%)	
Smoke, *n* (%)					0.008^**^
Yes	45 (32.6%)	79 (52.7%)	61 (43.0%)	56 (43.1%)	
No	93 (67.4%)	71 (47.3%)	81 (57.0%)	74 (56.9%)	
Alcohol, *n* (%)					0.176^**^
Yes	42 (30.4%)	59 (39.3%)	42 (29.6%)	31 (23.8%)	
No	96 (69.6%)	91 (60.7%)	100 (70.4%)	99 (76.2%)	
Site of brain trauma					
Frontal lobe, *n* (%)					0.032^**^
Yes	65 (47.1%)	52 (34.7%)			
No	73 (52.9%)	98 (65.3%)			
Temporal lobe, *n* (%)					0.001^**^
Yes	94 (68.1%)	57 (38.0%)			
No	44 (31.9%)	93 (62.0%)			
Parietal lobe, *n* (%)					0.705^**^
Yes	32 (23.2%)	32 (21.3%)			
No	106 (76.8%)	118 (78.7%)			
Occipital lobe, *n* (%)					0.428^**^
Yes	23 (16.7%)	20 (13.3%)			
No	115 (83.3%)	130 (86.7%)			
The severity of TBI					
Mild, *n* (%)					0.037^**^
Yes	16 (11.6%)	31 (20.7%)			
No	122 (88.4%)	119 (79.3%)			
Moderate, *n* (%)					0.043^**^
Yes	50 (36.2%)	72 (48.0%)			
No	88 (63.8%)	78 (52.0%)			
Serious, *n* (%)					0.001^**^
Yes	72 (52.2%)	47 (13.3%)			
No	66 (47.8%)	103 (86.7%)			
Surgical operation, *n* (%)					0.001^**^
Yes	47 (34.1%)	33 (22.0%)			
No	91 (65.9%)	117 (78.0%)			
Cause of brain trauma					
Traffic accidents, *n* (%)					0.118^**^
Yes	65 (47.1%)	57 (38.0%)			
No	73 (52.9%)	93 (62.0%)			
Falls, *n* (%)					0.503^**^
Yes	49 (35.5%)	59 (39.3%)			
No	89 (64.5%)	91 (60.7%)			
Strike the head, *n* (%)					0.265^**^
Yes	24 (17.4%)	34 (22.7%)			
No	114 (82.6%)	116 (77.3%)			

Remark:^*^ P-value extracted from Kruskal–Wallis test; ^**^ *P-*value extracted from chi-squared test

### Comparison of inflammatory parameters between groups

In the PTE group, both neutrophil count and NLR exhibited higher levels compared to the TBI group, the epilepsy of unknown origin group, and the HC group. Similarly, within the TBI group and the epilepsy of unknown origin group, the neutrophil count and NLR surpassed those in the HC group. Conversely, the lymphocyte count, platelet count, and PLR in the PTE group were lower in contrast to the TBI group, the epilepsy of unknown origin group, and the HC group. Correspondingly, in the TBI group and the epilepsy of unknown origin group, the lymphocyte count, platelet count, and PLR recorded lower values than those in the HC group.

The results showed significant differences between PTE and TBI, PTE and HC, TBI and HC, and epilepsy of unknown origin and HC in neutrophil count, lymphocyte count, platelet count, NLR and PLR (*P* < 0.05). Significant differences in neutrophil count, platelet count, NLR, and PLR between the PTE group and the epilepsy of unknown origin group (*P* < 0.05). However, there was no statistically significant difference in the lymphocyte counts (*P* > 0.05). These observations indicated that these inflammatory parameters varied significantly between different groups, as shown in Table 2.

**Table 2:** Between-group comparisons of NLR, PLR, and other inflammatory parameters among PTE, TBI, epilepsy of unknown origin, and healthy controls

Characteristics	Neutrophils	Lymphocytes	Platelets	NLR	PLR
PTE (*n* = 138)	5.09 ± 1.19	1.37 ± 0.43	128.6 ± 35.11	3.99 ± 1.34	98.87 ± 27.31
TBI (*n* = 150)	3.87 ± 0.93	1.49 ± 0.38	170.7 ± 50.98	2.78 ± 0.99	121.2 ± 41.95
Epilepsy of unknown origin (*n* = 142)	4.50 ± 1.23	1.73 ± 0.48	206.7 ± 57.83	2.72 ± 0.86	123.2 ± 31.89
Healthy control (*n* = 130)	3.42 ± 0.33	1.96 ± 0.52	261.7 ± 29.66	1.86 ± 0.51	142.8 ± 41.72
^*^ *P*	0.001	0.001	0.001	0.001	0.001
^* *^ *P*	0.001	0.114	0.001	0.001	0.001
^***^ *P*	0.001	0.001	0.001	0.001	0.001
^****^ *P*	0.001	0.001	0.001	0.001	0.001
^*****^ *P*	0.001	0.006	0.001	0.001	0.009

Remark: The* P*-values were all extracted from an independent Kruskal–Wallis test; ^*^ *P* is the group comparison between the PTE group and the TBI group; ^* *^P is the group comparison between the PTE group and the group with epilepsy of unknown origin; ^***^ *P* is for comparing the PTE group with the HC group; ^****^ *P* is the comparison of the TBI group and the HC group; ^*****^ *P* Comparison of the group with epilepsy of unknown origin with HC group

### Multivariate linear regression analysis of the influencing factors associated with the level of inflammation index in the PTE group

The outcomes of the clinical data, presented in Table 1, were employed as independent variables in the analysis. These variables encompass smoking, the location of brain trauma (frontal lobe and temporal lobe), the severity of TBI (mild, moderate, and severe), and surgical operation. Neutrophils, lymphocytes, platelets, NLR, and PLR served as dependent variables in the analysis. Utilising multivariate linear regression, an investigation was conducted to scrutinise the connection between these independent variables and the dependent variables in individuals affected by PTE. The objective of this analysis was to uncover potential correlations between clinical variables and inflammatory parameters in PTE patients.

The statistical outcomes suggest that the severity of TBI (mild, moderate, and severe) and surgical intervention are notable risk factors linked to neutrophil levels in individuals affected by PTE (*P* < 0.05). These findings are detailed in Table 3.

**Table 3:** Multivariate binary linear regression analysis of the relation with neutrophil levels in PTE patients

Characteristics	B	Standard error	Beta	*P*	The 95.0% confidence interval in B	
					Inferior limit	Superior limit
Smoking	−0.204	0.130	−0.082	0.120	−0.460	0.053
Brain trauma site						
Frontal lobe	0.104	0.129	0.042	0.419	−0.150	0.358
Temporal lobe	0.194	0.130	0.079	0.137	−0.062	0.451
The severity of TBI						
Mild	0.687	0.183	0.208	0.000	1.048	−0.327
Moderate	0.458	0.144	0.185	0.002	0.742	−0.175
Serious	0.853	0.140	0.343	0.000	1.128	0.825
Surgical operation	0.285	0.129	0.117	0.028	0.539	0.941

Remark: B: Unstandardized regression coefficients. Beta: Standardized regression coefficients. The *P*-values were obtained from the multiple linear regression

Similarly, the statistical outcomes show that the severity of TBI (mild, moderate, and severe) constitutes a noteworthy risk factor affecting lymphocyte levels in individuals with PTE (*P* < 0.05). This information is available in Table 4.

**Table 4:** Multivariate binary linear regression analysis of the relation with lymphocyte levels in PTE patients

Characteristics	B	Standard error	Beta	*P*	The 95.0% confidence interval in B	
					Inferior limit	Superior limit
Smoking	−0.042	0.040	−0.051	0.303	−0.121	0.038
Brain trauma site						
Frontal lobe	0.000	0.040	0.000	0.998	−0.078	0.078
Temporal lobe	−0.034	0.040	−0.042	0.393	−0.114	0.045
The severity of TBI						
Mild	−0.321	0.057	−0.292	0.000	−0.209	0.432
Moderate	−0.160	0.048	−0.195	0.001	0.067	−0.175
Serious	−0.355	0.043	−0.430	0.000	−0.440	−0.270
Surgical operation	−0.006	0.040	−0.008	0.877	−0.084	0.072

Remark: B: Unstandardized regression coefficients. Beta: Standardized regression coefficients. The *P*-values were obtained from the multiple linear regression

Moreover, the statistical outcomes indicate that the severity of TBI (severe) and surgical intervention are contributing factors affecting platelet levels in individuals with PTE (*P* < 0.05). These details are illustrated in Table 5.

**Table 5:** Multivariate binary linear regression analysis of the relation with platelet levels in PTE patients

Characteristics	B	Standard error	Beta	*P*	The 95.0% confidence interval in B	
					Inferior limit	Superior limit
Smoking	11.079	5.700	0.113	0.053	−0.142	22.299
Brain trauma site						
Frontal lobe	−10.401	5.634	−0.105	0.066	−21.492	0.690
Temporal lobe	−7.239	5.693	−0.074	0.205	−18.445	3.968
The severity of TBI						
Mild	14.302	8.004	0.109	0.075	−1.454	30.058
Oderate	7.301	5.812	0.074	0.210	−4.139	18.740
Serious	−13.308	6.104	−0.135	0.030	−25.323	−1.293
Surgical operation	−14.719	5.629	−0.151	0.009	−25.800	−3.639

Remark: B: Unstandardized regression coefficients. Beta: Standardized regression coefficients. The *P*-values were obtained from the multiple linear regression

Furthermore, the statistical outcomes suggest that the location of TBI (temporal lobe), along with the severity of TBI (mild, moderate, and severe) and surgical intervention, are notable risk factors linked to the NLR in individuals with PTE (*P* < 0.05). These results are detailed in Table 6.

**Table 6:** Multivariate binary linear regression analysis of the relation with NLR levels in PTE patients

Characteristics	B	Standard error	Beta	*P*	The 95.0% confidence interval in B	
					Inferior limit	Superior limit
Smoking	−0.304	0.160	−0.114	0.059	−0.619	0.011
Brain trauma site						
Frontal lobe	−0.146	0.157	−0.054	0.354	−0.455	0.163
Temporal lobe	0.432	0.154	0.164	0.006	0.128	0.735
The severity of TBI						
Mild	1.713	0.185	0.480	0.000	1.349	2.077
Moderate	1.000	0.146	0.375	0.000	0.712	1.288
Serious	1.972	0.107	0.736	0.000	1.761	2.182
Surgical operation	0.480	0.153	0.182	0.002	0.178	0.782

Remark: B: Unstandardized regression coefficients. Beta: Standardized regression coefficients. The *P*-values were obtained from the multiple linear regression

Lastly, the statistical outcomes indicate that the severity of TBI (moderate and severe) serves as a notable risk factor affecting the PLR in individuals with PTE (*P* < 0.05). These details are outlined in Table 7.

**Table 7:** Multivariate binary linear regression analysis of the relation with PLR levels in PTE patients

Characteristics	B	Standard error	Beta	*P*	The 95.0% confidence interval in B	
					Inferior limit	Superior limit
Smoking	10.599	4.277	0.141	0.104	2.180	19.017
Brain trauma site						
Frontal lobe	−8.945	4.227	−0.118	0.503	−17.267	−0.623
Temporal lobe	−3.375	4.271	−0.045	0.430	−11.784	5.033
The severity of TBI						
Mild	−9.561	6.005	−0.095	0.112	−21.383	2.260
Moderate	−1.000	0.146	−0.375	0.000	−1.288	−0.712
Serious	−20.957	4.580	−0.277	0.000	−29.972	−11.942
Surgical operation	−7.971	4.223	−0.107	0.060	−16.284	0.342

Remark: B: Unstandardized regression coefficients. Beta: Standardized regression coefficients. The* P*-values were obtained from the multiple linear regression

### The neutrophil, lymphocyte, platelet, NLR and PLR were analyzed by ROC curve

**Fig. 1:**
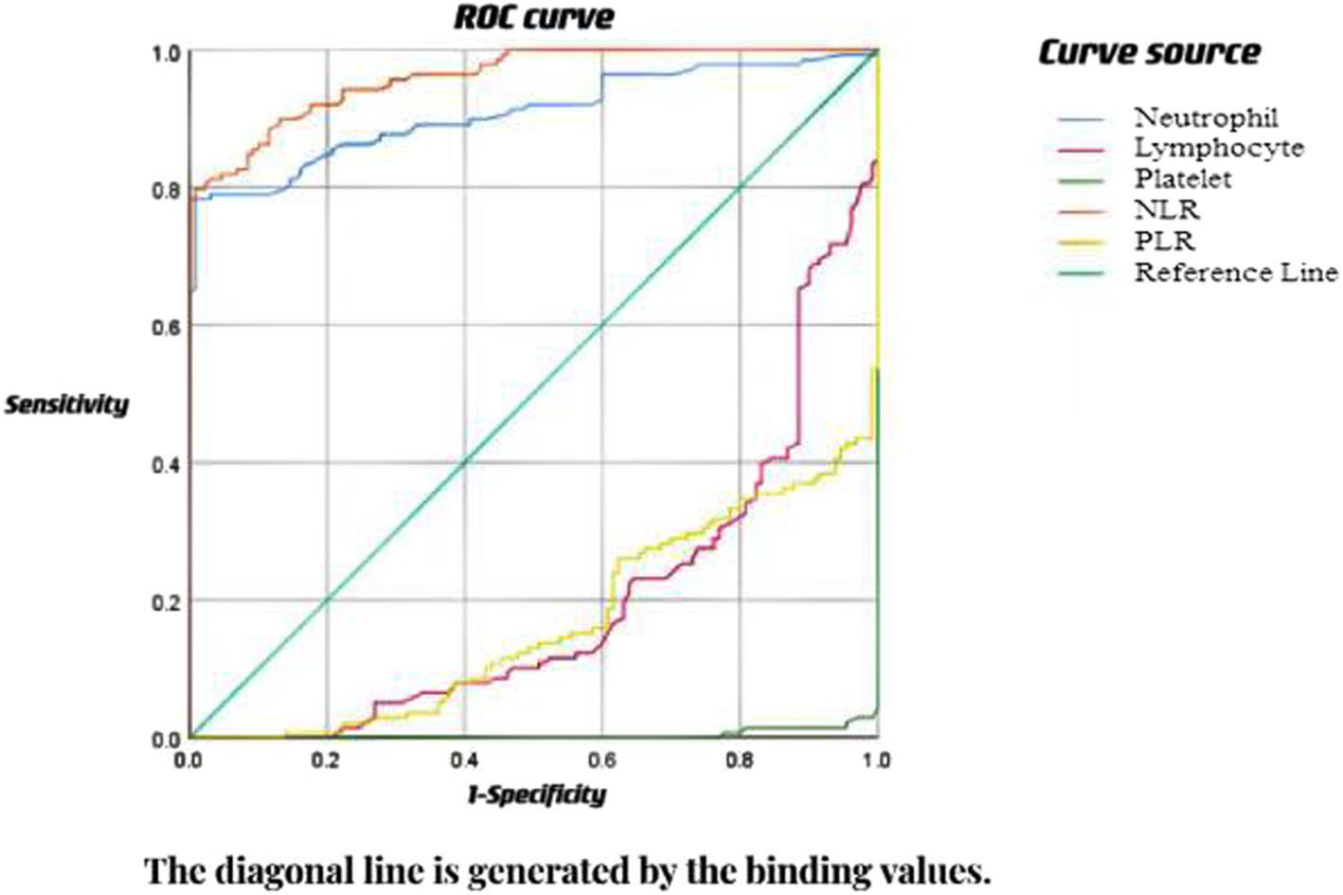
The neutrophil, lymphocyte, platelet, NLR and PLR were analyzed by ROC curve

The AUC values for lymphocytes, platelets, and the PLR were all below 0.5. However, for neutrophils, the identified optimal cut-off value was 4.05, yielding an AUC value of 0.908 (95% CI: 87.00%−94.50%). The test's sensitivity was computed as 0.783, and its specificity was determined to be 0.992. Similarly, the optimal cut-off value for the NLR was 2.945, resulting in an AUC value of 0.960 (95% CI: 94.0−98.0%). The NLR test exhibited a sensitivity of 0.797, with a specificity of 0.992. The results indicate that neutrophils, NLR may be a good predictor of PTE. These findings are graphically depicted in Fig. 1.

## Discussion

To our knowledge, this is the first report indicating significant differences in peripheral inflammation markers between PTE patients and healthy controls. Neutrophils and NLR may be the predictors of PTE. These findings offer new insights that could potentially contribute to the development of more effective strategies for preventing PTE.

In our investigation, we noted a noteworthy increase in neutrophil counts and NLR within the TBI cohort when contrasted with the healthy control cohort. In contrast, the TBI group exhibited reduced lymphocyte and platelet counts, as well as a diminished PLR compared to the HC group. A previous study showed that the NLR level was significantly higher than that in the control group [28], and Özdemir also found that the NLR level in the epilepsy group was higher than that in the healthy control group [29]. Reduced platelet count was an independent predictor of mortality in patients with TBI [30], which is in agreement with our findings.

The increase in neutrophil count and NLR may result from the following mechanisms: in the context of TBI, the release of pro-inflammatory cytokines from damaged meninges and parenchyma induces heightened extravasation of neutrophils, intensifying their response to injury [31]. Additionally, macrophages migrate to the CNS, initiating the activation of pro-inflammatory microglia and exacerbating ischemic brain injury, this process contributes to the observed reduction in lymphocyte count [32]. Consequently, the dysregulated immune response, characterized by heightened neutrophil activity and diminished lymphocyte count, results in an elevated NLR.

The reduction in PLR primarily occurs due to a decline in platelet count. However, in moderate to severe TBI cases, the balance between coagulation and anti-coagulation is disturbed. This disturbance can result in excessive platelet activation and a subsequent reduction in platelet numbers during the initial stages of injury [30, 33]. Consequently, the reduction in PLR mirrors the disrupted coagulation process and platelet activation observed in TBI. The platelet count decreases, contributing to the observed decline in PLR. Understanding these fundamental mechanisms helps elucidate the reason behind the PLR decrease in TBI patients.

This explains why neutrophils, NLR are increased, and lymphocytes, platelets, and PLR are decreased in TBI patients. A retrospective study by Ebner et al. found that in patients with ischemic stroke, the higher the NLR value, the higher the risk of early seizures after stroke. This biomarker seems to be useful in assessing the risk of early-onset seizures after stroke. This also underscores the potential role of inflammation in epileptogenesis [34]^.^ Therefore, they can be used as potential indicators of inflammation severity and poor prognosis in TBI patients.

Güneş et al. previously demonstrated significantly elevated levels of neutrophils, NLR, and PLR, along with markedly reduced levels of lymphocytes and platelets in epilepsy patients compared to controls [26]. However, in our investigation, we observed significant elevation only in neutrophil and NLR levels within the epilepsy group in comparison to the control group. These discrepancies from prior research may be ascribed to variations in the ethnic compositions of the study cohorts and the relatively confined sample sizes in each category. Ethnicity can impact immune responses and inflammatory markers, introducing variations in results among diverse populations. Moreover, the constrained sample sizes in individual studies may curtail the statistical power to discern significant differences for specific parameters.

Nonetheless, as of now, no specific peripheral blood inflammatory markers for PTE have been unearthed. To bridge this knowledge gap, we conducted a study delving into blood inflammatory parameters and computed the NLR and PLR. The deployment of NLR and PLR as tools for evaluating and prognosticating PTE holds the potential to introduce inventive approaches to clinical diagnosis and treatment strategies.

In our inquiry, we adopted a distinct approach by juxtaposing the PTE cohort against the TBI cohort, the epilepsy of unknown origin cohort, and the control cohort. Our findings showed that the PTE cohort displayed markedly elevated levels of neutrophils and NLR when contrasted with the TBI cohort, epilepsy of unknown origin cohort, and the control cohort. In contrast, levels of lymphocytes, platelets, and PLR were substantially diminished in the PTE cohort compared to the other cohorts.

This may be because the combination of brain trauma and epilepsy leads to a more aggressive inflammatory response in the body. The acute inflammatory response allows it to play a more prominent role in many aspects of PTE, including blood–brain barrier disorders, initiation and escalation of oxidative stress, and initiation and progression of cerebral oedema [35]. Nonetheless, it is crucial to note that a more extensive analysis and substantiation of these findings might necessitate a larger sample size and the incorporation of ethnically diverse groups.

Furthermore, our multivariate linear regression analysis unveiled that the TBI site (temporal lobe) (*P* < 0.05), the severity of TBI (mild,moderate,severe) (*P* < 0.05), and surgical intervention (*P* < 0.05) are the risk factors affecting the peripheral blood inflammation indicators.

TBI causes damage to the temporal lobe, which is well known for its susceptibility to epilepsy. The occurrence of temporal lobe epilepsy (TLE) is marked by the complex interaction of genetic and environmental factors [36–38]. There is increasing evidence confirming the involvement of inflammatory mechanisms in epilepsy, especially in TLE [39]. Previous studies have also shown increasing levels of inflammatory cytokines in epilepsy patients, particularly in the TLE cohort [40]. The results of this study suggest that damage to the temporal lobe is a risk factor for peripheral inflammatory markers, which confirms the previous findings. Emphasizing the significance of understanding the inflammatory mechanisms associated with temporal lobe damage and its possible consequences for epilepsy.

The results of this study suggest that the severity of TBI is also a risk factor for the development of PTE. Evidence exists as early as the early twentieth century: the likelihood of developing PTE escalates with the severity of traumatic brain injury. The 30-year cumulative incidence of epilepsy is 2.1% for mild TBI, 4.2% for moderate TBI, and 16.7% for severe TBI [41].

In addition, surgical intervention is also a risk factor for PTE development. Even small surgery on extra cranial tissue in mice induced a significant increase (300% increase) in granulocyte mobilisation and systemic cytokine induction [42, 43]. It has also been shown that surgical intervention can alter peripheral immune cell populations and may trigger systemic inflammation [40]. This study showed that surgery in the posterior part of the brain affects the inflammatory index in peripheral blood, which is consistent with earlier findings.

The ROC curve analysis revealed that, in patients with PTE, NLR stands out as the most reliable predictor. Furthermore, neutrophils were recognized as a moderately predictive factor. Nevertheless, PLR showed no discernible predictive value in this particular scenario.

The NLR is viewed as a mirror reflecting the intricate equilibrium between inflammatory and immune states within the body. An elevated NLR signifies a suppressed immune response mediated by lymphocytes [44]. Conversely, diminished levels of the PLR hint at an early disruption in coagulation equilibrium and increased neuroinflammatory activity [24]. In the realm of systemic inflammation, peripheral blood inflammatory markers play a crucial role, offering a direct, non-invasive, and easily accessible means of assessment.

Despite the absence of systematic investigations into the inflammatory state in PTE patients, our study identified significant variations in neutrophils, platelets, lymphocytes, NLR, and PLR across diverse groups. These observations hint at the potential of PTE to instigate a persistent inflammatory response, culminating in heightened NLR and diminished PLR, ultimately contributing to atypical systemic and local inflammatory reactions. This innovative perspective holds promise for impacting the clinical understanding and management of PTE. In comparison to alternative inflammatory markers, NLR stands out due to its simplicity, ease of measurement, and clinical applicability.

However, it's crucial to acknowledge the limitations inherent in our study, encompassing a constrained sample size and its retrospective nature as a single-centre investigation. Substantiating our findings might necessitate broader-scale randomized controlled clinical trials spanning multiple centres.

## Conclusions

To summarize, peripheral blood inflammatory markers, particularly NLR and PLR, offer valuable insights into the inflammatory landscape linked to PTE. While their simplicity and clinical relevance enhance their utility, additional research is imperative for validation and wider applicability.

## Supplementary Information

Additional file 1.

## Data Availability

All materials and data in this article are available. The original data can be found in the attachment.

## Abbreviations

BBB: Blood brain barrier
CNS: Central nervous system
CSF: Cerebrospinal fluid
CT: Computed Tomography
GCS: Glasgow coma score
HC: Healthy controls
MRI: Magnetic Resonance Imaging
NLR: Neutrophil-to-lymphocyte ratio
PLR: Platelet-to-lymphocyte ratio
PTE: Post-traumatic epilepsy
TBI: Traumatic brain injury
TLE: Temporal lobe epilepsy
